# No Overt Clinical Immunodeficiency Despite Immune Biological Abnormalities in Patients With Constitutional Mismatch Repair Deficiency

**DOI:** 10.3389/fimmu.2018.01506

**Published:** 2018-07-02

**Authors:** Victoria K. Tesch, Hanna IJspeert, Andrea Raicht, Daniel Rueda, Nerea Dominguez-Pinilla, Luis M. Allende, Chrystelle Colas, Thorsten Rosenbaum, Denisa Ilencikova, Hagit N. Baris, Michaela H. M. Nathrath, Manon Suerink, Danuta Januszkiewicz-Lewandowska, Iman Ragab, Amedeo A. Azizi, Soeren S. Wenzel, Johannes Zschocke, Wolfgang Schwinger, Matthias Kloor, Claudia Blattmann, Laurence Brugieres, Mirjam van der Burg, Katharina Wimmer, Markus G. Seidel

**Affiliations:** ^1^Research Unit Pediatric Hematology and Immunology, Division of Pediatric Hematology-Oncology, Department of Pediatrics and Adolescent Medicine, Medical University Graz, Graz, Austria; ^2^Department of Immunology, Erasmus MC, University Medical Center Rotterdam, Rotterdam, Netherlands; ^3^Hereditary Cancer Laboratory, University Hospital Doce de Octubre, i+12 Research Institute, Madrid, Spain; ^4^Department of Pediatric Hematology and Oncology, Virgen de la Salud Hospital, Toledo, Spain; ^5^i+12 Research Institute, University Hospital Doce de Octubre, Madrid, Spain; ^6^Department of Immunology, University Hospital Doce de Octubre, i+12 Research Institute, Madrid, Spain; ^7^Genetics Department, Curie Institute, Paris, France; ^8^Department of Pediatrics, Sana Kliniken Duisburg, Duisburg, Germany; ^9^Department of Pediatrics, Comenius University Bratislava, Bratislava, Slovakia; ^10^The Genetics Institute, Rambam Health Care Campus, The Ruth and Bruce Rappaport Faculty of Medicine, Technion – Israel Institute of Technology, Haifa, Israel; ^11^Pediatric Hematology and Oncology, Klinikum Kassel, Kassel, Germany; ^12^Pediatric Oncology Center, Department of Pediatrics, Technische Universität München, Munich, Germany; ^13^Department of Clinical Genetics, Leiden University Medical Center, Leiden, Netherlands; ^14^Department of Pediatric Oncology, Hematology and Transplantation, Poznań University of Medical Sciences, Poznań, Poland; ^15^Pediatrics Department, Hematology-Oncology Unit, Faculty of Medicine, Ain Shams University, Cairo, Egypt; ^16^Department of Pediatrics and Adolescent Medicine, Medical University of Vienna, Vienna, Austria; ^17^Division of Human Genetics, Medical University Innsbruck, Innsbruck, Austria; ^18^Department of Applied Tumor Biology, Institute of Pathology, Medical University Heidelberg, Heidelberg, Germany; ^19^Department of Hematology, Oncology, and Immunology, Olgahospital Stuttgart, Stuttgart, Germany; ^20^Department of Pediatric and Adolescent Oncology, Gustave Roussy Cancer Campus, Villejuif, France

**Keywords:** primary immunodeficiency, hyper-IgM syndrome, DNA repair defect, mismatch repair, somatic hypermutation, class-switch recombination, IgA deficiency, IgG subclass deficiency

## Abstract

Immunoglobulin class-switch recombination (CSR) and somatic hypermutations (SHMs) are prerequisites for antibody and immunoglobulin receptor maturation and adaptive immune diversity. The mismatch repair (MMR) machinery, consisting of homologs of MutSα, MutLα, and MutSβ (MSH2/MSH6, MLH1/PMS2, and MSH2/MSH3, respectively) and other proteins, is involved in CSR, primarily acting as a backup for nonhomologous end-joining repair of activation-induced cytidine deaminase-induced DNA mismatches and, furthermore, in addition to error-prone polymerases, in the repair of SHM-induced DNA breaks. A varying degree of antibody formation defect, from IgA or selective IgG subclass deficiency to common variable immunodeficiency and hyper-IgM syndrome, has been detected in a small number of patients with constitutional mismatch repair deficiency (CMMRD) due to biallelic loss-of-function mutations in one of the MMR genes (*PMS2, MSH6, MLH1, or MSH2*). To elucidate the clinical relevance of a presumed primary immunodeficiency (PID) in CMMRD, we systematically collected clinical history and laboratory data of a cohort of 15 consecutive, unrelated patients (10 not previously reported) with homozygous/compound heterozygous mutations in *PMS2* (*n* = 8), *MSH6* (*n* = 5), and *MLH1* (*n* = 2), most of whom manifested with typical malignancies during childhood. Detailed descriptions of their genotypes, phenotypes, and family histories are provided. Importantly, none of the patients showed any clinical warning signs of PID (infections, immune dysregulation, inflammation, failure to thrive, etc.). Furthermore, we could not detect uniform or specific patterns of laboratory abnormalities. The concentration of IgM was increased in 3 out of 12, reduced in 3 out of 12, and normal in 6 out of 12 patients, while concentrations of IgG and IgG subclasses, except IgG4, and of IgA, and specific antibody formation were normal in most. Class-switched B memory cells were reduced in 5 out of 12 patients, and in 9 out of 12 also the CD38^hi^IgM^−^ plasmablasts were reduced. Furthermore, results of next generation sequencing-based analyses of antigen-selected B-cell receptor rearrangements showed a significantly reduced frequency of SHM and an increased number of rearranged immunoglobulin heavy chain (IGH) transcripts that use IGHG3, IGHG1, and IGHA1 subclasses. T cell subsets and receptor repertoires were unaffected. Together, neither clinical nor routine immunological laboratory parameters were consistently suggestive of PID in these CMMRD patients, but previously shown abnormalities in SHM and rearranged heavy chain transcripts were confirmed.

## Introduction

Biallelic germline mutations in a mismatch repair (MMR) gene result in a condition referred to as *Constitutional Mismatch Repair Deficiency Syndrome* (CMMRD; OMIM #276300). This rare, devastating, cancer predisposition syndrome overlaps with the autosomal recessive form of Turcot’s syndrome, a condition characterized by the co-occurrence of multiple adenomatous colon polyps with an increased risk of colorectal cancer and of brain tumors (1). In addition, individuals with CMMRD have a very high risk of developing hematological and other malignancies starting in early childhood [reviewed in Ref. (2)]. Often, patients with CMMRD show café-au-lait macules (CALMs) and other signs reminiscent of neurofibromatosis type 1 (NF1) which is of diagnostic importance (3). For the clinical diagnosis of CMMRD and tumor surveillance in affected patients, recent consensus reports provide helpful diagnostic scores and screening guidelines (4–7).

The main function of the MMR system is repairing replication errors that escape the proofreading activity of the polymerases [reviewed in Ref. (8)]. In addition, the MMR system is involved (i) in immunoglobulin class-switch recombination (CSR) in that it recognizes activation-induced cytidine deaminase- (AID) catalyzed conversion of cytidines to uridines in DNA switch regions and (ii) in somatic hypermutation (SHM) [reviewed in Ref. (9)]. Both processes are needed for B cell maturation and for diversification and specification of the mammalian immunoglobulin repertoire. Defects of CSR are the molecular basis of hyper-IgM syndromes, which are primary immunodeficiencies (PIDs) with a predominant antibody formation defect associated with decreased IgG, IgA, and IgE, and normal or increased concentrations of IgM (9–11). With these functions, the MMR system constitutes a link between the immune system and tumor suppression (12).

Various levels of immunodeficiency were detected in single CMMRD patients or small patient series, supporting the hypothesis that the MMR machinery contributes to immunoglobulin CSR and SHM. IgA deficiency or common variable immunodeficiency (CVID) was first reported in one MSH2- and three MSH6-deficient patients (13–15). Further analyses focused on defects related to CSR and allowed the identification of three PMS2- and eight MSH6-deficient individuals with biallelic loss-of-function mutations, who presented variable degrees of hyper-IgM-like features and clear defects of CSR *in vitro* and *in vivo* (16, 17). In addition, larger screens for single nucleotide polymorphisms within MMR genes in selected patient cohorts with IgA deficiency or with CVID led to the identification of certain monoallelic *MSH5, MLH1*, and *MSH2* variants which could be linked to these PIDs (18, 19). Together, the results of these studies suggested that CMMRD consistently entails a PID.

The risk of malignancies is higher in most primary immune deficiency and dysregulation disorders (PID), but the mechanisms and frequencies of malignant transformation vary according to the different categories of PID (20). In CMMRD, any impairment of the immune system might be critical for the evolution of malignancies, since it would compromise tumor immune surveillance, which could accelerate tumorigenesis in addition to the remarkably increased mutation rates that are intrinsic to cells with MMR deficiency. Because previous studies reported varying degrees of immunodeficiency in patients with CMMRD that might render them less responsive to oncological immune therapy such as, e.g., checkpoint inhibition, the clarification of whether CMMRD patients suffered from PID has potential implications for future oncologic immune treatment strategies. On the other hand, a uniform pattern of clinical symptoms such as warning signs suggestive of PID or laboratory immunological abnormalities could facilitate early diagnosis of CMMRD. Furthermore, immunodeficiency secondary to chemotherapy might be aggravated in these individuals, requiring additional caution and supportive measures.

The present systematic analysis of PID in CMMRD addressed the *in vivo* cellular, humoral, and clinical immune phenotypes of CMMRD patients from Europe and the Middle East.

## Results

Fifteen consecutive, unrelated patients with a genetically confirmed diagnosis of CMMRD reported from nine countries were included in this study (11 females, 4 males; age at inclusion: 1–38 years, median age 9 years; age at first malignancy: 0.7–22 years, median age 5 years). Five of these patients were included in previous studies, while data of the remaining 10 patients were not published previously. Table 1 summarizes the patients’ genotypes, clinical presentations, and family histories.

**Table 1:** Characteristics of 15 patients with CMMRD.

Patient (reference)	Age (years) at study inclusion^a^	Genotype (mutated gene, mutation at cDNA level, mutation at protein level)	First symptom or malignancy (age, years)^b^; C4CMMRD points at first tumor diagnosis	History of clinical immunodeficiency or dysregulation^c^	Family tumors (age, years)	Parental consanguinity (as reported by parents)	Nonmalignant features	Premalignancies	Hematological malignoma (age, years)	Brain tumor (age, years)	LS-associated cancer (age, years)	Others
P1 (21)	38	PMS2 c.[137 G>T]; [137 G>T]; p.[Ser46Ile]; [Ser46Ile]	CRC (22); 6	Negative	LS family, mother: CRC (46)	Yes	Adenoma sebaceum, hepatic hemangioma	Dysplastic adenomata (colon)	–	Glioblastoma (34)	CRC (22) Duodenal Ca (36) Endometrial Ca (35)	

P2 (unpublished)	4	MSH6 c.[467C>G]; [1316A>G]; p.[Ser156*]; [Asp439Gly]	Anaplastic medulloblastoma (4); 5	Negative	Paternal grandfather: CRC (50), maternal cousin: AML^h^ (11)	No	CALM, freckling, ash-leaf spots; hemangioma, non-therapy-induced cavernoma		–	Anaplastic medulloblastoma (4)	–	–

P3 (22)	6	MSH6 c.[3261dupC]; [3261dupC]; p.[Phe1088Leufs*5]; [Phe1088Leufs*5]	CALM, T-NHL (3); 6	Negative	Two cousins affected with CMMRD-related malignancies	Yes	~10× CALM (generalized), bilateral frontal venous angioma; supra- and infratentorial hamartoma		T-NHL (3) T-NHL relapse [as T-ALL] (6)	–	–	–

P5 (unpublished)	10	PMS2 c.[634C>T]; [1239del]; p.[Gln212*]; [Asp414Thrfs*34]	Glioblastoma (9); 4	Negative	No	No	CALM		–	Glioblastoma (9)	–	–

P6 (23)	26	PMS2 c.[2192T>G]; [2192T>G]; p.[Leu731*]; [Leu731*]	CRC (20); 7	n.a.	Maternal grandfather: CRC (40)	Yes	CALM	Villous adenoma (small bowel)	–	Low grade diffuse astrocytoma (23) → high grade (26)^d^	CRC (20) Papilla Vateri Ca (22)	–

P7 (unpublished)	21	MLH1 c.[62C>A]; [2146G>A]; p.[Ala21Glu]; [Val716Met]^e^	T-NHL (1); 6	Negative	LS family, mother: CRC (40), maternal aunt: CRC (50), maternal grandfather: CRC (64)	No	1× CALM, cerebral cavernoma, varicosis, vascular malformation with pigmentation disorder right calf	9 adenomas (small, large bowel)	T-NHL (1) B-NHL (12)	Glioblastoma (21)	–	Borderline phylloides tumor (16)

P8 (unpublished)	1	MLH1 c.[332C>T]; [332C>T]; p.[Ala111Val]; [Ala111Val]	T-NHL (7 months); 6	Negative	Maternal grand-mother: CRC/breast cancer; paternal uncle: ColonCa (34)	Yes	CALM		T-NHL (7 months)	–	–	–

P9 (unpublished)	3	PMS2 c.[2007-2A>G]; [2007-2A>G]^f^	ALL (2); 6	Negative	Brother: CRC (12)	Yes	CALM		ALL (2)	Glioblastoma (3)	–	–

P10 (unpublished)	7	MSH6 c.[2653A>T]; [2653A>T]; p.[Lys885*]; [Lys885*]	Wilms tumor (5); 6	Negative	Maternal uncle: B-NHL; affected siblings-CMMRD	Yes	CALM		–	–	–	Wilms tumor (5)

P11 (24)	7	PMS2 c.[2444C>T]; [2444C>T]; p.[Ser815Leu]; [Ser815Leu]	CALM (7); n.a.	Negative	No	Yes	CALM		–	–	–	–

P12 (unpublished)	13	PMS2 c.[1515delG]; [1515delG]; p.[Phe506fs]; [Phe506fs]	B-cell Burkitt lymphoma (13); 6	Negative	No	No	CALM Venocapillary malformation	Multiple dysplastic colonic adenomatous polyps	B-cell Burkitt lymphoma (13)	–	–	–

P13 (unpublished)	10	MSH6 c.[1135_1139delAGAGA]; [2277_2293del]; p.[Arg379*]; [Glu760Profs*6]	B-ALL (3); 3	Negative	No	No	CALM (>6); Spitz naevus; hypopigmented areas; MRI signal alterations reminiscent of NF1-FASI; colitis chronica	Tubulous adenoma, low grade dysplasia	B-ALL; T-NHL (3; 7)	–	–	–

P14 (unpublished)	7	MSH6 c.[2238dupT]; [2980T>A]; p.[Leu747Serfs*9]; [Tyr994Asn]	Medulloblastoma (6); 3	Negative	No	No	CALM	–		Medulloblastoma (6 years)		

P15 (21)	12	PMS2 c.[2007-2A>G]; [2007-2A>G]^f^	T-NHL (4); 4	Negative	No	No	CALM (>5), hypopigmented macules	–	T-NHL (4)	Glioblastoma (9)		

P16 (unpublished)	9	PMS2 c.[1145-31_1145-13del]; [1145-31_1145-13del]; p.[Asn383*; Gly382Valfs*19]; [Asn383*; Gly382Valfs*19]^g^	Glioblastoma (8); 4	Negative	Paternal grandfather: prostate carcinoma (>70 years); one sister died from NHL (4 years)	Yes	– (no signs of NF1)			Glioblastoma (8)		

*^a^At one time point within 4 years of patient recruitment, when blood sampling for immunological analyses was undertaken*.

*^b^At first malignancy*.

*^c^Defined by immunological warning signs as assessed by a questionnaire (Figure S1 in Supplementary Material) with data shown in Table 2*.

*^d^Counted as one malignancy*.

*^e^p.Val716Met is classified a benign variant according to the InSiGHT Variant Interpretation Committee and it cannot be excluded that this patient carries a different pathogenic mutation on this allele*.

^f^In a different patient, it was shown that the mutation c.2007-2A>G leads to the following two aberrant transcripts: r.2007_2023del (p.Ser669Argfs*9) and r.2007_2174del (p.Ser669_Ala725delinsArg).

^g^cDNA-sequencing showed two aberrant transcripts: r.1144_1145insGATAGTCCACGTTTGCTTAG (p.Asn383Ter) and r.1145_2006del (p.Gly382Valfs*19).

*^h^In this family member, a germline mutation in CEBPa associated with a predisposition toward myeloid malignancies was detected*.

*CRC, colorectal carcinoma; LS, Lynch syndrome; CALM, café-au-lait macule (spots); T-NHL, T-cell Non-Hodgkin’s Lymphoma; ALL, acute lymphoblastic leukemia; NF1-FASI, NF1-associated foci of abnormal signal intensity; CMMRD, constitutional mismatch repair deficiency*.

In line with previous observations, homozygous (*n* = 7) or compound heterozygous (*n* = 1) *PMS2* germline mutations were present in more than half of the patients; consanguinity was reported by five of the parents (Table 1). Two of the four novel patients with PMS2-deficiency (P5 and P12) had truncating mutations affecting both *PMS2* alleles and the other two (P9 and P16) were homozygous for splice mutations leading to aberrant out-of-frame transcripts. Six of PMS2-deficient patients had a recent history of high-grade malignant glioma, and one had a recent history of Burkitt’s lymphoma. In one patient with glioblastoma, acute lymphoblastic leukemia (ALL), and in another one, T-cell Non-Hodgkin’s lymphoma (T-NHL) had preceded the brain tumor by 1 and 5 years, respectively. Two patients had metachronous LS-associated carcinomas, and three patients also had bowel adenomas.

Five patients had *MSH6* mutations. Two patients from reportedly consanguineous parents were each homozygous for a truncating *MSH6* mutation. Three patients were compound heterozygous for two different *MSH6* mutations. Interestingly, one of these patients (P13) had a *de novo* mutation that was absent in both genetically confirmed parents, while the second mutation was maternally inherited. While this patient had two different truncating mutations, the other two patients (P2 and P14) had one truncating and one missense mutation. Both missense *MSH6* mutations (p.Asp439Gly and p.Tyr994Asn) are so far unreported, but could be classified as likely pathogenic at least in the context of CMMRD according to ACMG guidelines (25).The tumor spectrum of MSH6-deficient patients included Wilms tumor, two medulloblastomas, and two NHL, which relapsed in one patient and was preceded by a B-cell ALL in the other patient (Table 1). One of the MSH6-deficient patients had bowel adenomas at 10 years of age. With a median age of 6.5 (range 4–7) years, none of the other patients had bowel adenomas or LS-associated tumors.

One of two patients with biallelic mutations in *MLH1* was from consanguineous parents. This patient (P8) carried the known missense mutation p.Ala111Val classified as likely pathogenic by the InSiGHT Variant Interpretation Committee. The other patient (P7) was compound heterozygous for the known missense mutation p.Ala21Glu classified as pathogenic by the InSiGHT Variant Interpretation Committee and the missense variant p.Val716Met which is classified as benign variant by the InSiGHT Variant Interpretation Committee. Of note, both patients (P7 and P8) showed in a germline microsatellite instability (gMSI) assay elevated gMSI ratios as did all PMS2-deficient individuals tested for this feature and which is highly specific for CMMRD (26). Although, we cannot exclude that not the detected variant p.Val716Met but a different *MLH1* mutation is responsible for CMMRD in patient P7, it is noteworthy, that this variant has already previously been discussed to be potentially responsible in combination with a stop-mutation for the colorectal cancer in a 12-year-old boy (27). Both MLH1-deficient patients (P7 and P8) had T-NHL at a very early age (8 months and 1 year), and P7 developed another lymphoma (B-NHL) at 12 years of age, a borderline phylloides tumor at 16 and a glioblastoma at 21. She also had multiple bowel adenomas removed.

Two patients (P3 and P10) were from families with previously diagnosed CMMRD patients. One patient (P11) was diagnosed with CMMRD prior to tumor development (24). All 12 remaining index patients in the families fulfilled the C4CMMRD criteria for the clinical suspicion of CMMRD when they had their first tumor with a mean of 5.5 (range 3–7) C4CMMRD scoring points (5). Non-malignant features indicative of CMMRD according to the C4CMMRD scoring system were present in 13 out of 15 patients. Two or more CALM, hyper- and/or hypopigmented skin patches were noted in 13 patients, and cerebral cavernoma/hamartoma were present in three. Furthermore, vascular anomalies of the skin (hemangioma and venocapillary malformation) were reported in three patients, and one patient had a hepatic hemangioma. Parents of eight patients reported consanguinity. Family histories of LS or LS-associated carcinomas in the first, second, or third degree relatives were reported for five patients, and three had siblings with CMMRD-associated cancers (Table 1).

To define the clinical immunodeficiency associated with CMMRD, we first assessed the clinical immunological parameters. Patients’ clinical history data were obtained using a study questionnaire, which is provided in the Online Repository (Figure S1 in Supplementary Material; see Patients and Methods). Data were retrieved from 14 out of 15 patients and showed no clinical signs of PID (Table 2). A filled questionnaire was not available from P6, but her physician-reported history did not show any signs of PID. Only 2 out of 14 patients (P2 and P3; aged 4 and 6 years) were reported to have 4–7 infections of the ear-nose-throat tract or simple viral infections per year, which is within the physiological range at that age (Table 2). For none of the other patients an increased number of infections were reported. P3 had a history of bronchial asthma, with an IgE within the normal range (Table 2).

**Table 2:** History of clinical signs of immunodeficiency or immune dysregulation of 14 (out of 15)^a^ patients with CMMRD.

	P1	P2	P3	P5	P7	P8	P9	P10	P11	P12	P13	P14	P15	P16
**Immunodeficiency warning signs**														
Family history of immunodeficiency	No	No	No	No	No	No	No	No	No	No	No	No	No	No
Hospitalization for infections	No	No	Only during chemo	No	No	Only during chemo	Only during chemo	Only during chemo	No	No	No	Yes (in Syria)	No	No
Reactions/complications after live vaccines	No	No	No	No	No	n.a.	No	No	No	No	No	No	No	n.d.
Pathological healing of the navel	No	No	No	No	No	No	No	No	No	No	No	n.a.	No	n.d.
Delayed growth during childhood/reduced thriving	No	No	No	No	No	No	No	No	No	No	No	No	No	No

**Infections**														
Frequency (per year)	0	7	4–5	Normal	Normal	Increased since chemo	Increased since chemo	Increased during chemo	Normal	Normal	Normal	1	Normal	Normal
Severity	n.a.	Simple viral	Simple viral, simple bacterial; history of bronchial asthma	Simple viral	Simple viral	Complicated viral complicated bacterial	Invasive fungal	Simple viral and invasive fungal	n.a.	n.a.	n.a.	Complicated viral	Simple viral	Simple viral
Localization	n.a.	Respiratory tract	ENT, bacterial: lungs	ENT	ENT	Lungs bacteremia	Skin	n.d.	n.a.	n.a.	n.a.	Bone marrow, liver	n.a.	n.a.
Infectious agents	n.a.	n.d.	During chemo: *Staphylococcus aureus*, MRSA, *Aspergillus fumigatus*	n.d.	Normal spectrum	During chemo: opportunistic	During chemo: opportunistic, *Candida albicans*	During chemo: Herpes simplex, *C. albicans*	n.a.	n.a.	n.a.	Parvovirus HCV	n.a.	n.a.
Response to antibiotics	n.a.	Normal	Delayed, during chemo	n.a.	Normal	Delayed	n.d.	Normal	n.a.	Normal	Normal	Normal	n.a.	n.a.
Requirement of corticosteroids or immunosuppression	Only as oncological treatment	No	Yes, during chemo	No	No	Yes, during chemo	n.d.	No	No	Yes, during chemo	No	No	n.a.	No

**Autoimmunity/immune dysregulation**														
Granuloma	No	No	No	No	No	No	No	No	No	No	No	No	No	No
Autoimmunity	No	No	No	No	No	No	No	No	No	No	No	No	No	No
Autoinflammation/recurrent fever	No	No	No	No	No	No	No	No	No	No		No	n.a.	No
Lymphoproliferation/splenomegaly	No	No	Yes, mild axillary	No	No	No	No	No	No	No	Yes	No	No	No
Hepatopathy, cholangitis, *Cryptosporidium* infection	No	No	No	No	No	No	No	No	No	No	No	No	No	No
Inflammatory bowel disease	No	No	No	No	No	No	No	No	No	No	No	No	No	No
Interpretation regarding primary immunodeficiency (PID)	No signs	No signs	No signs	No signs	No signs	No signs	No signs	No signs	No signs	No signs	No signs	No signs	No signs	No signs

*^a^No immunological history data available from patient P6*.

*ENT, ear-nose-throat tract; chemo, cytostatic chemotherapy; MRSA, multidrug resistant Staphylococcus aureus; CMMRD, constitutional mismatch repair deficiency*.

Next, we sought to determine whether there was any consistent abnormality detectable in the routine parameters of the cellular immune system. To this end, an analysis resembling an extended, routine, diagnostic workup of any suspected, combined immunodeficiency was performed. Due to the role of the MMR machinery in CSR and SHM, we focused on the B cell maturation stages. But also, T cell subsets and the T cell receptor repertoire were analyzed. Tables 3 and 4 show the raw data for the most relevant B and T cell subsets together with the corresponding normal ranges (28, 29). Patients are grouped according to their genotype (i.e., mutated MMR gene) and sorted according to age. Importantly, no uniform pattern of variation from the norm was identified within the cellular immune system of CMMRD patients as a result of the routine PID diagnostic FACS analysis, including an analysis of memory B cell subsets. Class-switched memory B cells were reduced to a varying degree in 5 and normal in 7 out of 12 patients. Still, we detected a clear trend of reduced CD38^hi^IgM^−^ plasmablasts (measured as a percent of B cells in 12 patients: normal in 3 patients, reduced in 7, absent in 2, and an increased in none) and a relative increase of CD21^low^CD38^low^ (activated) B cells in 6 of 12 patients across all three genotypes analyzed (Table 3). As expected, values of the following lymphocyte subsets were unremarkable with mild, inconsistent variations: T cells and T cell subsets including CD4^+^ and CD8^+^ T cells, T cell receptor alpha/beta (TCRab)-positive CD4^−^CD8^−^CD3^+^ double negative T cells, TCRgamma/delta as well as naïve CD45RA^+^CD4^+^ (Table 4) and naïve CD8^+^ T cells (not shown), activated T cells (not shown), and NK cells and monocytes (Table 4). Furthermore, the results of the TCR repertoire (spectratyping), assessed by conducting quantitative sequencing of 24 TCR Vbeta family fragments from CD4 and CD8 T cells, was unremarkable in all individuals tested (*n* = 14; not shown). Overall, apart from a reduction in the number of class-switched B memory cells in 5 out of 12 individuals and of class-switched plasmablasts detected in most (9 out of 12), no abnormality of the cellular immune system was consistently found among these CMMRD patients.

**Table 3:** Quantification of B cell subsets of CMMRD patients.

	CD19/μL	CD19^+^CD27^+^IgD^+^ (ncsBm) %B	CD19^+^CD27^+^IgD^−^ (csBm) %B	CD19^+^CD27^−^IgD^+^(naive) %B	CD21^lo^CD38^lo^ (activ) %B	CD38^hi^IgM^hi^ (trans) %B	CD38^hi^IgM^−^ (csPlasmablasts) %B
*P9 (PMS2; 3 years)^a^*	*7*	–	–	–	–	–	–
P11 (PMS2; 7 years)	548	15.21	4.52	73.87	8.29	5.6	**0.15**
P16 (PMS2; 8 years)	**213**	7.31	10.47	76.92	6.32	8.07	0.53
P5 (PMS2; 10 years)	**193**	13.85	9.88	74.75	6.84	8.22	**0**
P12 (PMS2; 13 years)	**775**	5.31	7.30	78.54	**22.32**	4.24	0.45
P15 (PMS2; 14 years)	**168.5**	5.1	5.37	80.81	**69.43**	4.7	**0.14**
P6 (PMS2; 26 years)	**1,079**	**3.3**	**4.67**	**84.29**	**32.54**	1.39	**0**
P1 (PMS2; 38 years)	317	**6.75**	**1.93**	**89.29**	**84.21**	**0.73**	**0.1**
P2 (MSH6; 4 years)	**57**	**0.35**	**1.06**	**93.47**	**80.91**	5.78	**0.35**
*P3 (MSH6; 6 years)*	*46.5*	*10*	*30*	*0*	*6.67*	*0*	*0*
P10 (MSH6; 7 years)	**179**	3.21	5.02	**90.54**	5.14	20.09	0.65
P14 (MSH6; 7 years)	582	4.71	5.54	84.49	9.25	7.99	**0.23**
P13 (MSH6; 10 years)	**195**	**1.95**	**2.26**	93.46	8.02	3.61	**0.04**
*P8 (MLH1; 1 year)^a^*	***8***	*8*	*4*	***80***	–	–	–
P7 (MLH1; 21 years)	342	**3.15**	**2.14**	**92.24**	**62.95**	1.29	**0.18**
**Controls/reference values (5th–95th percentile)**							
1 year^b^ (*n* = 26)	700–1,300	3.25–10.75	1–5	83.25–93.75	1–11	1–25	0.4–3.6
2–3 years^b^ (*n* = 38)	700–1,300	4.9–14.2	2.9–9.2	74.7–90.5	1–11	1–25	0.4–3.6
4–5 years^b^ (*n* = 38)	700–1,300	7–15.2	3.9–16.2	69.9–85.6	1.11	1–25	0.4–3.6
6–10 years^b^ (*n* = 38)	300–500	2.93–19	3.85–16.5	63.1–89.15	1–11	1–25	0.4–3.6
11–18 years^b^ (*n* = 22)	300–500	5.05–17.95	4–22.8	60.15–88.95	1–11	1–25	0.4–3.6
19–61 years^b^ (*n* = 54)	300–500	7.4–32.5	6.5–29.1	42.6–82.3	1–11	1–25	0.4–3.6

*Samples are sorted according to gene defect and age*.

*^a^Data for patients P3, P8, and P9 were excluded from B cell analyses due to their recent treatment with rituximab (P8 and P9) and/or chemotherapy (P8 and P3) (*italic*)*.

*^b^Reference values for ncsBm, csBm, and naïve B-cells were taken from Huck et al. (29) for the age groups 1 year, 2–3 years, 4–5 years, 6–10 years, 11–18 years; and from Warnatz and Schlesier (28) for the age group 19–61 years and csPlasmablasts*.

*Reduced values are printed in **red boldface***.

*Increased values are printed in **green boldface***.

*CMMRD, constitutional mismatch repair deficiency*.

**Table 4:** Quantification of T cell subsets and other peripheral blood mononuclear cells of CMMRD patients.

	CD3^+^/μL	CD3^+^CD4^+^	CD3^+^CD8^+^	CD4^+^CD45RA^+^ (%CD3)	TCRab^+^CD4^−^CD8^−^CD56^−^ (%CD3)	TCRgd^+^ (%CD3)	NK/μL	Mono/μL	Stem cells in PB/μL
		
Age-specific normal range^a^	1,400–2,000	700–1,100	600–900	>11–30% (age-dep.)	<2%	<11–15% (age-dep.)	200–300	400–1,000	
P9 (PMS2; 3 years)	1,839 [1,800–3,000]	1,088 [1,000–1,800]	613 [800–1,500]	36.64	0.09	3.17	252	**2,254**	13
P11 (PMS2; 7 years)	**5,139**	**3,209**	**1,748**	53.81	0.06	7.21	566	609	3
P16 (PMS2; 8 years)	**1,161**	**529**	606	18.68	0.05	10.82	337	**323**	4
P5 (PMS2; 10 years)	**954**	**543**	**377**	77.22	0.20	5.93	**140**	400	1
P12 (PMS2; 13 years)	**2,864**	**1,361**	**1,258**	31.01	0.23	5.98	247	**185**	0
P15 (PMS2; 14 years)	1,786	**112**	755	30.25	0.13	5.37	278	684	0
P6 (PMS2; 26 years)	**2,481**	**1,485**	**948**	n.d.	n.d.	n.d.	**189**	**1,664**	14
P1 (PMS2; 38 years)	**2,053**	**1,342**	639	21.75	n.d.	1.7	266	**320**	n.d.
P2 (MSH6; 4 years)	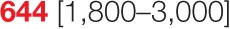	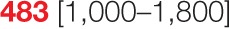	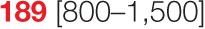	60.8	0.15	3.29	**124**	735	0
*P3 (MSH6; 6 years)*	*833*	*266*	*556*	*32.79*	*0*	*26.21*	*276*	*398*	*n.d*.
P10 (MSH6; 7 years)	**1,123**	**405**	681	36.30	0.20	7.10	**120**	427	1
P14 (MSH6; 7 years)	1,697	903	754	22.54	0.52	4.69	253	**386**	1
P13 (MSH6; 10 years)	**454**	**216**	**196**	**9.55**	0.12	3.52	**72**	444	1
P8 (MLH1; 1 year)^b^	71 [1,800–3,000]	53 [1,000–1,800]	15 [800–1,500]	39.93	0	5.41	24	374	2
P7 (MLH1; 21 years)	**1,378**	776	**538**	19.91	0.21	3.41	**323**	690	1

*Samples are sorted according to gene defect and age*.

*^a^Except otherwise stated [square brackets]*.

*^b^Data of patients P3 and P8 were excluded from T cell analyses due to preceding chemotherapy (italic)*.

*Reduced values are printed in **red boldface***.

*Increased values are printed in **green boldface***.

*TCRab, T cell receptor alpha/beta; CMMRD, constitutional mismatch repair deficiency*.

**Figure 1:**
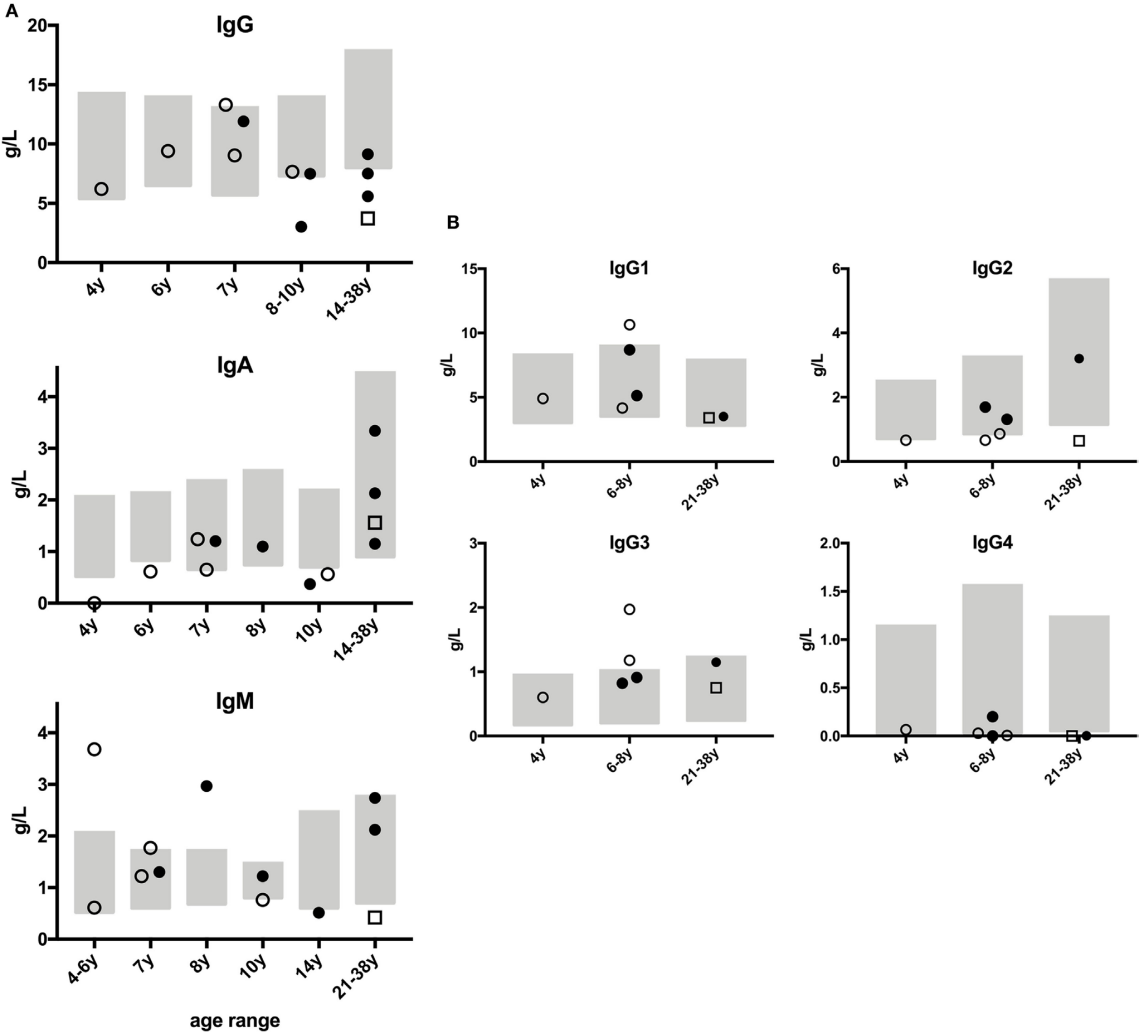
Immunoglobulin concentrations of patients with constitutional mismatch repair deficiency. Humoral immunologic analyses were performed locally at the patients’ hospitals, and the results were sent to the study center. None of the patients had received therapeutic (i.v. or s.c.) immunoglobulins within the last 6 months prior to analysis. Results from PMS2-deficient patients are shown as full dots, from MSH6-deficient patients as open dots, and from MLH1-deficient patient as open square. Age-specific normal ranges are shown as gray boxes. **(A)** Panels show serum concentrations of IgG, IgA, and IgM in g/dl, with age-specific, normal ranges. **(B)** Graphs show IgG subclass analyses, available from seven patients, related to age-specific, normal ranges to facilitate interpretation. Out of 15 patients, no serological data were available for P12, and those for P8 and P9 were excluded due to their having received prior chemo- or rituximab therapy.

Abnormal parameters of humoral immunity and B cell function such as IgG subclass or IgA deficiency, hypogammaglobulinemia, and hyper-IgM syndrome are expected in CMMRD as they were shown to occur in patients with defective CSR (16, 30, 31). Thus, we examined the results of the quantitative immunoglobulin isotypes and subclass analyses (Figure 1) and of the specific antibody formation capacity (Table S1 in Supplementary Material). These serological analyses were conducted at the patients’ local hospitals, and they were not conducted for all patients. The results were quite heterogeneous, with mostly normal, but some reduced and some increased immunoglobulin concentrations. Importantly, we did not observe a consistent reduction of IgA, IgG, or any IgG subclass except for IgG4 (in four out of seven tested patients). However, the interpretation of IgG4 subclass deficiency is limited, because normal ranges indicate that IgG4 might be undetectable throughout preschool age and still very low (0.05 g/l) even in healthy adults (32). IgG was mildly to moderately reduced in 4 out of 12 tested patients, all of whom were ≥10 years of age (Figure 1A). IgM was increased in 3 out of 12 of the tested patients (P2, P14, P16; Figure 1A). Of the three patients with increased IgM, one (P2) also had IgA deficiency and two (P2 and P14) a borderline reduction of IgG2. The third patient (P16) had remarkably increased IgM, but normal IgA and IgG subclass, values. P14, in whom IgM concentrations were only slightly increased, displayed a remarkable increase in IgG1 and IgG3 (Figure 1). Of note, three patients had reduced concentrations of IgM (P7, P13, P15; Figure 1A), and six individuals had IgM concentrations within the normal range. IgE was detectable within the normal range in three and nearly absent or undetectable in four individuals (not shown). Antibody concentrations against vaccination antigens or childhood infections (such as against diphtheria toxin, tetanus toxoid, hepatitis B virus, rubeola, morbilli, haemophilus influenza B polysaccharide, pneumococcal polysaccharide, varicella zoster virus, Epstein–Barr virus, cytomegalovirus; without recent therapeutic administration of immunoglobulins) tested positive for at least some of the tested antibodies in all patients (Table S1 in Supplementary Material). Of note, all three patients with increased IgM concentrations had detectable levels of specific antibody formation against protein and polysaccharide antigens (P2, P14, P16; Table S1 in Supplementary Material). Despite the limitation that the vaccination and infection histories were not assessed in detail, compromising the interpretation of specific antibody concentrations, these results do not support a specific antibody formation defect in CMMRD patients. On the contrary, patients with a known history of infection or current infection at the time of analysis displayed an adequately increased concentration of specific antibodies (Table S1 in Supplementary Material). The results of the attempt to detect antinuclear antibodies were inconclusive with three patients showing negative results and two (albeit asymptomatic) patients who tested low-level positive. Together, these data do not support the hypothesis that patients with CMMRD regularly have a clinically relevant humoral immunodeficiency.

**Figure 2:**
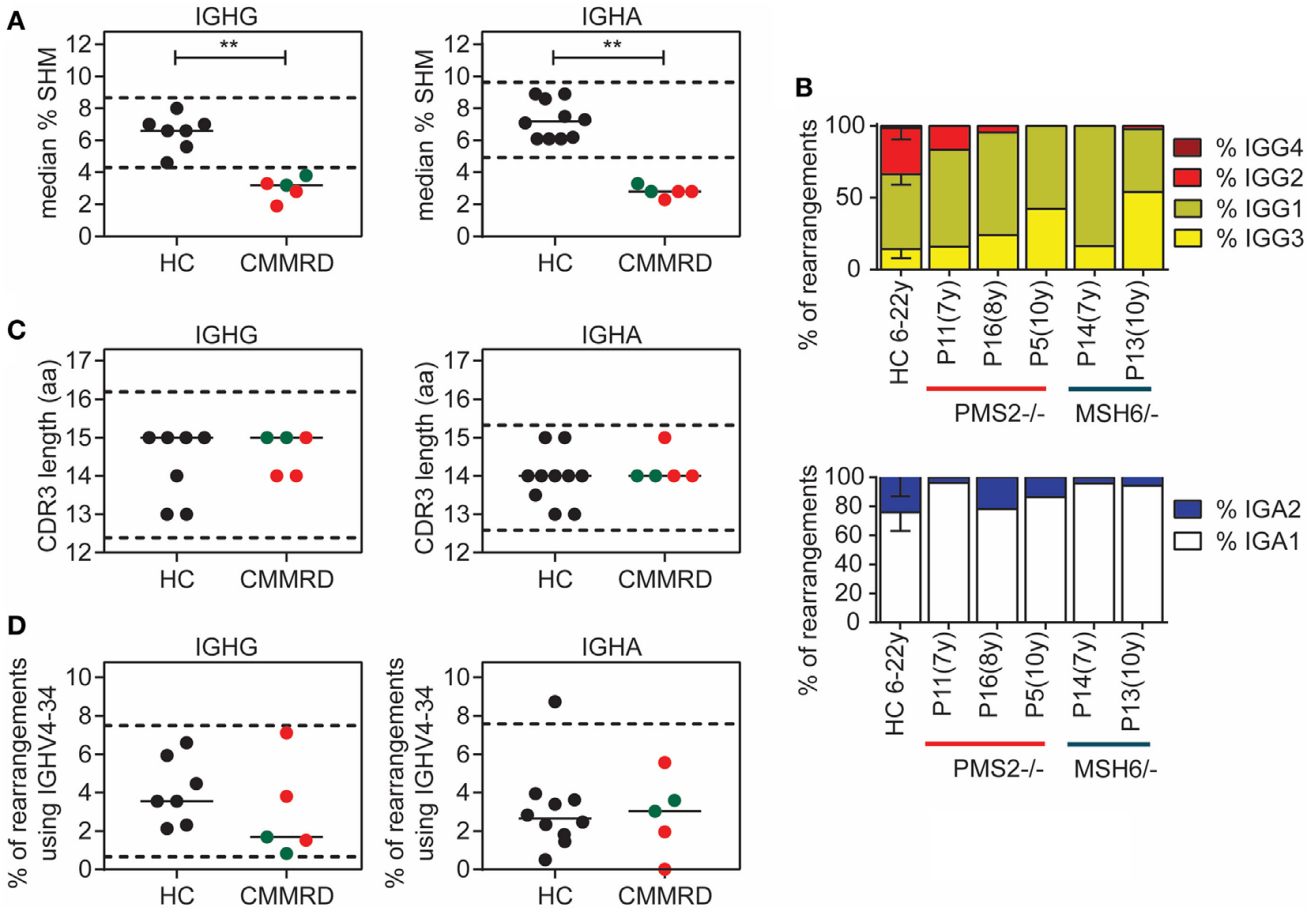
Analysis of somatic hypermutation (SHM) and class-switch recombination (CSR) in B-cell receptor rearrangements. Detailed analysis of B-cell receptor rearrangements showed significantly decreased frequency of SHM in patients with constitutional mismatch repair deficiency (CMMRD) compared to healthy controls (HC) **(A)**. In addition, the frequency of rearrangements that have the IGHG3, IGHG1, and IGHA1 subclass were increased in the CMMRD patients, suggesting a possible defect in CSR **(B)**. Selection against B cells with a long complementary determining region (CDR3) **(C)**, and B-cell that use the IGHV4-34 gene **(D)** was normal in the CMMRD patients. Red dots indicate MSH6-deficient patients and green dots indicate the PMS2-deficient patients. **P < 0.01 and ***P < 0.0005.

After B cells have encountered antigen, they migrate to the germinal center where they undergo SHM, CSR, and selection. To study the effect of MMR deficiency on these processes at the molecular level, we analyzed the B-cell receptor repertoire in five CMMRD patients using next generation sequencing of IGHG and IGHA transcripts derived from antigen-selected B cells. We obtained 253–568 unique immunoglobulin heavy chain (IGH) rearrangements per patient and compared them to age-matched healthy controls (HC) (Table S4 in Supplementary Material). Interestingly, the median frequency of SHM was significantly reduced in both IGHG and IGHA transcripts in all patients (Figure 2A). In addition, we analyzed the subclass distribution in the IGHG and IGHA transcripts. This distribution in the IGHG transcripts was altered in four of the five CMMRD patients, who hardly had IGHG transcripts that used the IGHG2 or IGHG4 subclasses (Figure 2B). Also, in the IGHA transcripts, fewer transcripts with the IGHA2 subclass were present in four of the five CMMRD patients as compared to the HC. The IGHG2 and IGHG4 constant genes are located more distal in the IGH locus, further away from VDJ rearrangement compared to the IGHG3 and IGHG1 constant gene, and are used during sequential switching during a single immune response or after consecutive germinal center response. A reduction in these subclasses is often seen in patients with a CSR defect, like patients with Ataxia telangiectasia (33), and might indicate a defect in CSR or a disturbed immune response. Given the fact that the frequency of SHM is also reduced it is likely that B-cells in germinal center fail to undergo a second round of affinity maturation and therefore do not make it until switching toward the distal constant regions [see also Ref. (34)].

When B cells differentiate from naïve B cells to memory B cells, they are strongly selected against B cells that have a long complementary determining region (CDR3), and against B cells that express the IGHV4–34 gene, because they are both associated with autoreactive B cells (35, 36). In the CMMRD patients, both the CDR3 length and the frequency of rearrangements that use the IGHV4–34 gene was normal (Figures 2C,D), suggesting that the selection against autoreactive B cells is normal and not affected by the MMR deficiency.

## Discussion

This study included 10 hitherto unpublished and 5 recently reported, unrelated patients with CMMRD. Overall, the genotypes and clinical presentations of these 15 patients, with respect to the pattern of malignancies, age-of-onset, and the spectrum of non-malignant symptoms, are in agreement with previous findings. Interestingly, two of the patients had extended skin areas with vascular malformations, a feature that has not (yet) been included in the C4CMMRD scoring system (5). Patient P10 is the second CMMRD patient who has been reported with Wilms tumor (nephroblastoma), and patient P7 is the first CMMRD patient with a reported phylloides tumor. With respect to potential genotype–phenotype correlations, it is noteworthy that two MSH6-deficient patients (P2 and P14) had a medulloblastoma. Because six of the ten previously published CMMRD cases with medulloblastoma were also MSH6-deficient, and given that only approximately 20% of CMMRD patients are due to biallelic *MSH6* mutations, this suggests an over-representation of medulloblastoma among MSH6-deficient individuals. A high-grade glioma was diagnosed in seven patients, six of whom carried biallelic *PMS2* mutations. Among the patients with *PMS2* mutations, two (P1 and P6) had been diagnosed with colorectal cancer as first malignancy only in their early twenties, supporting the notion of less penetrant forms of CMMRD especially in PMS2-deficient individuals. Consistent with a higher penetrance of biallelic *MLH1* mutations, both patients with MLH1 deficiency (P7 and P8) had T-NHL in infancy. Nevertheless, one of them (P7) survived this as well as three additional, malignant tumors. We also report here the family history and data for the first CMMRD patient (P13) with one inherited and one *de novo MSH6* mutation which is absent in both parents.

In the present study, we tested whether CMMRD is associated with a clinically relevant PID with a predominant antibody deficiency or hyper-IgM-like syndrome and a consistent impairment of B memory formation, readily detectable by clinical history taking, physical examination, and routine immunological analyses. This hypothesis was based on the known role of the MMR machinery in the immunoglobulin CSR and SHM in humans and mice (8, 9, 11, 18, 37–44), and previous findings in CMMRD patients (13–17). In contrast to earlier studies, in which MMR genes and their function were examined in selected PID patient cohorts, we took a more clinical and unbiased approach, attempting to confirm and describe or exclude a manifest and relevant PID in a series of consecutively registered patients with CMMRD. Following this systematic analysis, only inconsistent laboratory abnormalities, partially recapitulating previous human and mouse data, were found within extended routine analyses of the cellular and humoral immune system, and these lacked any clinical correlation in terms of a symptomatic PID syndrome. None of the 15 patients showed any of the clinical warning signs of PID according to the international guidelines (45, 46). Although 3 out of 12 tested patients showed mildly to moderately increased IgM concentrations (1.77–3.68 g/dl with widely varying age-specific normal ranges), their IgG and specific antibody formation was intact, and only two of them had an accompanying borderline IgG2 reduction and one, an IgA deficiency. On the other hand, 3 out of 12 patients had decreased IgM concentrations, and most showed normal IgG and IgG subclass concentrations except IgG4. Unfortunately, a complete immunological laboratory analysis was performed in only 12 out of 15 patients. Recent chemo- or immunosuppressive therapy prevented the laboratory analyses in three patients, and the extended serological data could not be obtained from the patient’s local hospital in one patient. Although hyper-IgM syndrome cannot be excluded on mere humoral parameters, clearly, the majority of the analyzed patients did not have serological laboratory results that suggested hyper-IgM syndrome (e.g., increased IgM and simultaneously decreased IgG and/or IgA) or a symptomatic, specific antibody formation defect. The fact that IgG concentrations appeared to be reduced more frequently in adolescents and young adults (from 10 years of age and above; Figure 1A, upper panel) might indicate an age-dependent subclinical aggravation of the laboratory immune phenotype. By contrast, IgA concentrations were higher in these individuals than in younger patients (Figure 1A, center panel). These findings are too inconsistent to support, but might be in line with, the hypothesis that long-lived IgG- and/or IgA-producing plasma cells gained relevance with age in this context (9, 47). Furthermore, the number of patients is too little to confirm this observed trend of age-dependence or to suspect a genotype–phenotype correlation, especially since older patients in our study cohort included more individuals with PMS2 deficiency than individuals with other MMR deficiencies. Accordingly, a potential conclusion of a higher degree of immunodeficiency in PMS2-deficient as compared to MSH6-deficient patients, as suggested by the findings in previous studies showing that three out of nine PMS2-deficient patients were symptomatic and needed IgG substitution, as compared to none out of eight MSH6-deficient individuals (16, 17), cannot be corroborated by our data. Because our study comprised an unselected collection of patients consecutively registered with CMMRD within a given time frame, we can exclude a selection bias toward or against immunodeficiency. Also, due to the recruitment modality, an environmental or epigenetic bias is unlikely in the presented cohort. Nevertheless, despite the lack of a clinical correlate such as bacterial infections that could be ascribed to antibody deficiency in our cohort, our data confirm previous studies insofar as a proportion of CMMRD patients had immune biological abnormalities such as reduced class-switched and non-class-switched memory B cells and varying alterations in immunoglobulin subtypes, and we detected decreased numbers of class-switched CD38^hi^IgM^−^ plasmablasts in most individuals, which is together indicative for a sub-optimal germinal center reaction.

The data suggest a more redundant role for single components of the MMR system *in vivo* or for the MMR machinery as a whole in human, switched isotype, specific antibody formation, and B cell differentiation. These components could be substituted, e.g., by the base excision repair pathway (9, 11), rather than by other DNA repair mechanisms such as the MRE11–RAD50–NBS1 complex or by ATM and NHEJ (10). The findings in this cohort of CMMRD patients are reminiscent of the immune phenotype of patients with XRCC4 deficiency. XRCC4 is a binding partner of LIG4 and component of the NHEJ pathway. XRCC4-deficient individuals show a junctional immunoglobulin diversification defect but have normal immunoglobulin concentrations and lack clinical signs of immunodeficiency (48).

Autoimmune diseases are typical findings in patients with combined immunodeficiency syndromes including CD40/CD40L deficiency (hyper-IgM syndromes type 3 and 1, respectively) and in predominant antibody formation disorders such as CVID. Furthermore, systemic lupus erythematosus (SLE) is a frequent finding in a subgroup of complement deficiencies (49) and SLE has been previously described in at least three independent MSH6-deficient patients (17, 50, 51). We detected antinuclear antibodies in two of the five tested individuals in this cohort (P6, 26 years old and P11, 7 years old), both PMS2-deficient and asymptomatic with regards to autoimmunity. Due to the relatively high prevalence of antinuclear antibody (ANA) positivity (>10%) in the general pediatric and adolescent populations (52) and the small number of patients with CMMRD who have been tested for ANA to date, these findings should not be overvalued. Nevertheless, the association between CMMRD and ANA positivity and the risk of developing SLE should be further investigated, and at least a baseline ANA screening should be considered from the age of adolescence and onward.

Mismatch repair plays an important role in the resolution of the uracil:guanine mismatches that are introduced during SHM and CSR. Although on a cellular level no differences were found in the B-cell compartment, detailed molecular analysis of B-cell receptor rearrangements derived from antigen-selected B cells showed a clear reduction in the frequency of SHM and alterations in the IGHG and IGHA subclass distribution. These effects on SHM and CSR can have multiple causes, like a defect in T:B cell interaction, or an intrinsic defect in the SHM and CSR process itself. Based on the previous studies showing that MMR has a role in SHM and CSR, it is most likely that the defect observed in the CMMRD patients in SHM and CSR is caused by an intrinsic defect in these processes. This, however, did not result in constant changes in the frequency of memory B cells, and only in 3 out of 15 patients the level of IgM was mildly to moderately increased.

Taken together with previous findings our results suggest that, although IgG2/4 subclass deficiency, IgA deficiency, or—rarely—more severe phenotypes of antibody formation, B cell class switch, maturation, and memory formation defects may be found in patients with CMMRD, they are neither constant nor obligatory diagnostic hallmarks of this syndrome and tend to lack a clinical correlate.

## Patients and Methods

### Patient and Data Acquisition

Patient identification was achieved through the network of human geneticists and (pediatric) oncologists working throughout Europe and the Middle East who were informed about the study at conferences and *via* personal communication from 2014 to 2017. Most of participating physicians were partners of the consortium “Care for CMMRD (C4CMMRD).” Of the 19 patients who were originally included in the analysis as they were evaluated for CMMRD on the basis of their phenotypic and oncologic features, four had to be excluded since CMMRD was excluded. Two of these patients had a diagnosis of Lynch syndrome (each one had a heterozygous mutation in *PMS2* and *MSH2*) and two had other cancer prone conditions. Results from three patients had to be excluded from some of the analyses because they had recently received chemo- and/or B cell-depleting (anti-CD20) immune therapy (P3, P8, and P9, respectively). Importantly, in all other patients, blood sampling was undertaken before chemo-, steroid or immunosuppressive treatment or until an adequate interval after therapy, with readiness to ensure immune reconstitution, reflected in part by normal complete blood counts (not shown), monocyte, NK, and T cell subset analyses (Table 3), and confirmed by the physician in charge prior to lab analyses. None of the patients had received therapeutic immunoglobulins prior to inclusion and blood sampling. To obtain clinical history data with a focus on immunodeficiency, we designed a questionnaire interrogating the most relevant facts regarding the patients’ histories and clinical statuses including infections according to the “extended clinical warning signs for PID” (45, 46), inpatient or intravenous antibiotic treatment, failure to thrive, signs of immune dysregulation such as autoimmunity and inflammation, etc. (Figure S1 in Supplementary Material). Extended routine immunologic laboratory data were obtained by conducting retrospective chart reviews guided by the study questionnaire and by collecting results from recommended immunological analyses that were justified by previous reports on a varying degree of humoral immunodeficiency and impaired B cell maturation in defects of CSR (16, 17, 30, 31, 53). The study was performed in compliance with current guidelines for good clinical practice and the Declaration of Helsinki with an IRB approval (29-178 ex 16/17) from the Medical University Graz (IRB00002556).

### Genetic Laboratory Analyses

Standard molecular genetic testing included for all novel patients fully or partially analyzed at the Division of Human Genetics at the Medical University Innsbruck (P2, P7, P12, P13, P14, and P16) mutation analysis and concomitantly gMSI analysis of peripheral blood lymphocyte DNA as previously described by Ingham et al. (26). Increased gMSI ratios indicate biallelic mutations in *PMS2, MLH1*, or *MSH2*, while biallelic *MSH6* mutations escape the detection by this assay. For mutation analysis, all exonic coding and flanking intronic regions of the MMR and the EPCAM gene were enriched by using hybridization-based TruSightCancer panel (Illumina) and sequenced on a MiSeq platform (Illumina). Sequence data were analyzed with the SeqNext Software (JSI) and all variants present in ≥5% of the reads were classified according to the consensus recommendations of the American College of Medical Genetics (25) as (likely) pathogenic (denoted mutations), unclassified, or (likely) irrelevant variants. Quantitative analysis of the sequence data with respect to copy number variations (CNVs) was performed with the CNV tool of the SeqNext Software (JSI) and in parallel with the CNV Detective Software cnMOPS (54). CMMRD patients with a variant of unknown significance (VUS; or a monoallelic mutation) in one of the MMR genes were additionally analyzed by direct cDNA-sequencing of the entire coding sequence to assess for potential splice effects of VUS and/or to uncover/exclude (other) mutations that escaped the detection of massive parallel sequencing (55). This analysis was so far not possible for patient P7. For the detection of *PMS2* CNVs multiplex ligation-dependent probe amplification (MLPA) analysis using the SALSA MLPA-Kit P008-B1 (MRC Holland) was performed according to Wernstedt et al. (56).

Mutation analysis for patients P8, P9, and P10 was performed at the Hereditary Cancer Laboratory at the University Hospital Doce de Octubre (Madrid, Spain). Here, all exonic coding and flanking intronic regions of the MMR genes were amplified using a custom designed primer panel with Ion AmpliSeq Library Kit 2.0 reagent (ThermoFisher) and sequenced on an Ion PGM System (ThermoFisher). Data were analyzed using Ion Reporter software (ThermoFisher).

All detected mutations were confirmed in a second DNA-sample that was extracted from an independently extracted blood sample. Nucleotide positions were numbered according to the recommendations of the Human Genome Variation Society (57) with the A of the start codon ATG in exon 1 representing the nucleotide position c.1 using the reference numbers NM_000249.3 for *MLH1*, NM_000251.2 for *MSH2*, NM_000179.2 for *MSH6*, and NM_000535.5 for *PMS*2.

### Immunologic Laboratory Analyses

In addition to routine chemistry and clinical immunology laboratory analyses, special immunologic analyses were performed centrally in Graz, Austria, which included flow cytometry performed on a Cytomics FC500 flow cytometer (Beckman Coulter, Brea, Calif) with a panel of mAbs from Beckman Coulter (Vienna, Austria), Becton Dickinson (Vienna, Austria), Dako (Glostrup, Denmark), and Miltenyi Biotech (Vienna, Austria and Bergisch Gladbach, Germany). Analysis of TCR V beta diversity (spectratyping) was performed as follows: RNA from enriched subsets was extracted using RNeasy Protect Mini Kit (Qiagen, Hilden, Germany) following manufacturer instructions. The amount of RNA was determined with Eppendorf Biophotometer plus (Eppendorf, Hamburg, Germany). Finally, a concentration of 1 µg RNA was used for reverse transcription with a First Strand cDNA Synthesis Kit for RT-PCR AMV (Roche, Vienna, Austria), carried out following manufacturer instructions. cDNA was diluted 1:5 for PCR using AmpliTaq Gold™ DNA Polymerase (Applied Biosystems, Vienna, Austria), 1× PCR Gold Buffer (Applied Biosystems, Vienna, Austria), 2.5 mM MgCl_2_ (Applied Biosystems, Vienna, Austria), 0.4 mM dNTP Polymerization Mix (GE Healthcare, Vienna, Austria), 0.5 µM TCR C β 5′FAM labeled primer (Ingenetix, Vienna, Austria), and 0.5 µM unlabeled TCR V β primer (Ingenetix, Vienna, Austria) according to Monteiro et al. (58). This resulted in 25 reactions per sample. Cycle conditions were a denaturation step at 94°C for 6 min, 35 cycles at 94°C for 1 min each, 59°C for 1 min, and 72°C for 1 min, with a final annealing step at 72°C for 7 min. After amplification, 1 µl of PCR-product was supplemented with 0.5 µl of GeneSCan™-500 ROX™ Size Standard (Applied Biosystems, Vienna, Austria) and 12 µl HI-DI Formamide (Applied Biosystems, Vienna, Austria). Electrophoresis was performed with a 3130 Genetic Analyzer (Applied Biosystems, Vienna Austria) and 3130 Data Collection Software. Analyses were conducted using the GeneScan^®^ Software (Applied Biosystems, Vienna Austria). Calculations included peak count (Complexity score) and single peak area as percentages of whole peak area.

### Next Generation Sequencing of the B-Cell Repertoire

Peripheral blood mononuclear cells were isolated from peripheral blood using Ficoll, and mRNA was isolated using the Gen-Elute Mammalian total RNA miniprep kit from Sigma Aldrich (St. Louis, MO, USA). cDNA was created from 2 μg RNA using the Superscript II reverse transcriptase kit from Invitrogen (Paisley, UK). IGH transcripts were amplified in a multiplex PCR using the forward VH1-6 FR1 (BIOMED-2) primers and either the CgCH or the IGHA reverse primer which were adapted with a multiplex identifier sequence to be able to multiplex the PCR products (59–61). PCR products were purified by gel extraction (Qiagen, Valencia, CA, USA) and Agencourt AMPure XP beads (Beckman Coulter, Fullerton, CA, USA). The PCR products were sequenced on the 454 GS junior using the Lib-A V2 kit (Roche). The raw data were demultiplexed, 40 nt trimmed at the 5′ and 3′ side to remove the primer sequence, and converted to fasta files using ARGalaxy (62). The fasta files were uploaded in IMGT High-V-Quest (version 1.5.6) (63) for alignment with the reference sequences. Subsequently, the IMGT output files were analyzed using ARGalaxy (62). To obtain unique rearrangements and reduce the presence of errors in the sequences, only sequences present two or more times (based on CDR1–CDR3 nucleotide sequence) were included once in the analysis. In addition, incomplete sequences or sequences containing an ambiguous “*n*” base were excluded. Since the data from the CMMRD patients were very clonal (determined using Change-O) (64), we only included one sequence per clone in the analysis. Samples that contained less than 45 unique IGH rearrangements were excluded from the analysis. Data from the CMMRD patients was compared to 10 HC (6–22 years of age), which were previously published (36). Details on the number of sequences obtained after filtering can be found in Table S2 in Supplementary Material.

### Data Presentation

Due to the small patient number, only a descriptive data analysis was performed. Figures were designed using Prism 7.0c (GraphPad software, La Jolla, CA, USA).

## Ethics Statement

The study was performed in compliance with current guidelines for good clinical practice and the Declaration of Helsinki with an IRB approval (29-178 ex 16/17) from the Medical University Graz (IRB00002556). Extended routine immunologic laboratory data were obtained by conducting retrospective chart reviews guided by the study questionnaire and by collecting results from recommended immunological analyses that were justified by previous reports on a varying degree of humoral immunodeficiency and impaired B cell maturation in defects of class-switch recombination (16, 17, 30, 31, 53).

## Author Contributions

MS and KW designed the study. KW, CC, and LB organized patient identification and study inclusion. VT wrote the first draft of the manuscript and the tables; MS designed the figures. MS and KW edited the final version of the manuscript, which all authors approved to. MS, KW, and VT organized the documentation of clinical and laboratory data and the transfer of blood samples to (reference) laboratories. MS, AR, WS, MK, HI, and MB designed and performed immunological laboratory analyses that were not conducted locally. KW, SW, and JZ organized and performed molecular genetic tests that were not performed locally and reference analyses. DR, CC, TR, DI, HI, HB, MN, MS, DJ-L, IR, AA, CB, ND-P, LA, and LB cared for the patients, recorded their medical histories, collected laboratory data, and are active partners of the “Care 4 CMMRD” network.

## Conflict of Interest Statement

The authors declare that the research was conducted in the absence of any commercial or financial relationships that could be construed as a potential conflict of interest.

## References

[1] Hamilton SR Liu B Parsons RE Papadopoulos N Jen J Powell SM The molecular basis of Turcot’s syndrome. N Engl J Med (1995) 332:839–47. 10.1056/NEJM199503303321302 7661930

[2] Wimmer K Etzler J. Constitutional mismatch repair-deficiency syndrome: have we so far seen only the tip of an iceberg? Hum Genet (2008) 124:105–22. 10.1007/s00439-008-0542-4 18709565

[3] Wimmer K Rosenbaum T Messiaen L. Connections between constitutional mismatch repair deficiency syndrome and neurofibromatosis type 1. Clin Genet (2017) 91:507–19. 10.1111/cge.12904 27779754

[4] Vasen HF Ghorbanoghli Z Bourdeaut F Cabaret O Caron O Duval A Guidelines for surveillance of individuals with constitutional mismatch repair-deficiency proposed by the European consortium "care for CMMR-D" (C4CMMR-D). J Med Genet (2014) 51:283–93. 10.1136/jmedgenet-2013-102238 24556086

[5] Wimmer K Kratz CP Vasen HF Caron O Colas C Entz-Werle N Diagnostic criteria for constitutional mismatch repair deficiency syndrome: suggestions of the European consortium ‘care for CMMRD’ (C4CMMRD). J Med Genet (2014) 51:355–65. 10.1136/jmedgenet-2014-102284 24737826

[6] Durno C Boland CR Cohen S Dominitz JA Giardiello FM Johnson DA Recommendations on surveillance and management of biallelic mismatch repair deficiency (BMMRD) syndrome: a consensus statement by the US multi-society task force on colorectal cancer. Gastroenterology (2017) 152:1605–14. 10.1053/j.gastro.2017.02.011 28363489

[7] Tabori U Hansford JR Achatz MI Kratz CP Plon SE Frebourg T Clinical management and tumor surveillance recommendations of inherited mismatch repair deficiency in childhood. Clin Cancer Res (2017) 23:e32–7. 10.1158/1078-0432.CCR-17-0574 28572265

[8] Jiricny J. Postreplicative mismatch repair. Cold Spring Harb Perspect Biol (2013) 5:a012633. 10.1101/cshperspect.a012633 23545421 PMC3683899

[9] Durandy A Kracker S. Immunoglobulin class-switch recombination deficiencies. Arthritis Res Ther (2012) 14:218. 10.1186/ar3904 22894609 PMC3580555

[10] Davies EG Thrasher AJ. Update on the hyper immunoglobulin M syndromes. Br J Haematol (2010) 149:167–80. 10.1111/j.1365-2141.2010.08077.x 20180797 PMC2855828

[11] de la Morena MT. Clinical phenotypes of hyper-IgM syndromes. J Allergy Clin Immunol Pract (2016) 4:1023–36. 10.1016/j.jaip.2016.09.013 27836054

[12] de Miranda NF Bjorkman A Pan-Hammarstrom Q. DNA repair: the link between primary immunodeficiency and cancer. Ann N Y Acad Sci (2011) 1246:50–63. 10.1111/j.1749-6632.2011.06322.x 22236430

[13] Whiteside D Mcleod R Graham G Steckley JL Booth K Somerville MJ A homozygous germ-line mutation in the human MSH2 gene predisposes to hematological malignancy and multiple cafe-au-lait spots. Cancer Res (2002) 62:359–62. 10.1111/j.1399-0004.2007.00803.x 11809679

[14] Ostergaard JR Sunde L Okkels H. Neurofibromatosis von Recklinghausen type I phenotype and early onset of cancers in siblings compound heterozygous for mutations in MSH6. Am J Med Genet A (2005) 139A:96–105; discussion 196. 10.1002/ajmg.a.30998 16283678

[15] Scott RH Mansour S Pritchard-Jones K Kumar D Macsweeney F Rahman N. Medulloblastoma, acute myelocytic leukemia and colonic carcinomas in a child with biallelic MSH6 mutations. Nat Clin Pract Oncol (2007) 4:130–4. 10.1038/ncponc0719 17259933

[16] Peron S Metin A Gardes P Alyanakian MA Sheridan E Kratz CP Human PMS2 deficiency is associated with impaired immunoglobulin class switch recombination. J Exp Med (2008) 205:2465–72. 10.1084/jem.20080789 18824584 PMC2571921

[17] Gardes P Forveille M Alyanakian MA Aucouturier P Ilencikova D Leroux D Human MSH6 deficiency is associated with impaired antibody maturation. J Immunol (2012) 188:2023–9. 10.4049/jimmunol.1102984 22250089

[18] Sekine H Ferreira RC Pan-Hammarstrom Q Graham RR Ziemba B De Vries SS Role for Msh5 in the regulation of Ig class switch recombination. Proc Natl Acad Sci U S A (2007) 104:7193–8. 10.1073/pnas.0700815104 17409188 PMC1855370

[19] Offer SM Pan-Hammarstrom Q Hammarstrom L Harris RS. Unique DNA repair gene variations and potential associations with the primary antibody deficiency syndromes IgAD and CVID. PLoS One (2010) 5:e12260. 10.1371/journal.pone.0012260 20805886 PMC2923613

[20] Hauck F Voss R Urban C Seidel MG. Intrinsic and extrinsic causes of malignancies in patients with primary immunodeficiency disorders. J Allergy Clin Immunol (2018) 141:59–68.e54. 10.1016/j.jaci.2017.06.009 28669558

[21] Lavoine N Colas C Muleris M Bodo S Duval A Entz-Werle N Constitutional mismatch repair deficiency syndrome: clinical description in a French cohort. J Med Genet (2015) 52(11):770–8. 10.1136/jmedgenet-2015-103299 26318770

[22] Ilenčíková D Constitutional mismatch repair-deficiency syndrome (CMMR-D) - a case report of a family with biallelic MSH6 mutation. Klin Onkol (2012) 25(Suppl):S34–8. 22920205

[23] Baris HN Barnes-Kedar I Toledano H Halpern M Hershkovitz D Lossos A Constitutional Mismatch Repair Deficiency in Israel: High Proportion of Founder Mutations in MMR Genes and Consanguinity. Pediatr Blood Cancer (2016) 63(3):418–27. 10.1002/pbc.25818 26544533

[24] Suerink M Potjer TP Versluijs AB Ten Broeke SW Tops CM Wimmer K Constitutional mismatch repair deficiency in a healthy child: on the spot diagnosis? Clin Genet (2018) 93:134–7. 10.1111/cge.13053 28503822

[25] Richards S Aziz N Bale S Bick D Das S Gastier-Foster J Standards and guidelines for the interpretation of sequence variants: a joint consensus recommendation of the American College of Medical Genetics and Genomics and the association for molecular pathology. Genet Med (2015) 17:405–24. 10.1038/gim.2015.30 25741868 PMC4544753

[26] Ingham D Diggle CP Berry I Bristow CA Hayward BE Rahman N Simple detection of germline microsatellite instability for diagnosis of constitutional mismatch repair cancer syndrome. Hum Mutat (2013) 34:847–52. 10.1002/humu.22311 23483711

[27] Marcos I Borrego S Urioste M Garcia-Valles C Antinolo G. Mutations in the DNA mismatch repair gene MLH1 associated with early-onset colon cancer. J Pediatr (2006) 148:837–9. 10.1016/j.jpeds.2006.01.009 16769400

[28] Warnatz K Schlesier M. Flowcytometric phenotyping of common variable immunodeficiency. Cytometry B Clin Cytom (2008) 74:261–71. 10.1002/cyto.b.20432 18561200

[29] Huck K Feyen O Ghosh S Beltz K Bellert S Niehues T. Memory B-cells in healthy and antibody-deficient children. Clin Immunol (2009) 131:50–9. 10.1016/j.clim.2008.11.008 19162556

[30] Imai K Catalan N Plebani A Marodi L Sanal O Kumaki S Hyper-IgM syndrome type 4 with a B lymphocyte-intrinsic selective deficiency in Ig class-switch recombination. J Clin Invest (2003) 112:136–42. 10.1172/JCI18161 12840068 PMC162294

[31] Peron S Pan-Hammarstrom Q Imai K Du L Taubenheim N Sanal O A primary immunodeficiency characterized by defective immunoglobulin class switch recombination and impaired DNA repair. J Exp Med (2007) 204:1207–16. 10.1084/jem.20070087 17485519 PMC2118580

[32] Schauer U Stemberg F Rieger CH Borte M Schubert S Riedel F IgG subclass concentrations in certified reference material 470 and reference values for children and adults determined with the binding site reagents. Clin Chem (2003) 49:1924–9. 10.1373/clinchem.2003.022350 14578325

[33] Driessen GJ Ijspeert H Weemaes CM Haraldsson A Trip M Warris A Antibody deficiency in patients with ataxia telangiectasia is caused by disturbed B- and T-cell homeostasis and reduced immune repertoire diversity. J Allergy Clin Immunol (2013) 131:1367–75.e1369. 10.1016/j.jaci.2013.01.053 23566627

[34] Berkowska MA Driessen GJ Bikos V Grosserichter-Wagener C Stamatopoulos K Cerutti A Human memory B cells originate from three distinct germinal center-dependent and -independent maturation pathways. Blood (2011) 118:2150–8. 10.1182/blood-2011-04-345579 21690558 PMC3342861

[35] Wardemann H Yurasov S Schaefer A Young JW Meffre E Nussenzweig MC. Predominant autoantibody production by early human B cell precursors. Science (2003) 301:1374–7. 10.1126/science.1086907 12920303

[36] IJspeert H Van Schouwenburg PA Van Zessen D Pico-Knijnenburg I Driessen GJ Stubbs AP Evaluation of the antigen-experienced B-cell receptor repertoire in healthy children and adults. Front Immunol (2016) 7:410. 10.3389/fimmu.2016.00410 27799928 PMC5066086

[37] Ehrenstein MR Neuberger MS. Deficiency in Msh2 affects the efficiency and local sequence specificity of immunoglobulin class-switch recombination: parallels with somatic hypermutation. EMBO J (1999) 18:3484–90. 10.1093/emboj/18.12.3484 10369687 PMC1171427

[38] Schrader CE Edelmann W Kucherlapati R Stavnezer J. Reduced isotype switching in splenic B cells from mice deficient in mismatch repair enzymes. J Exp Med (1999) 190:323–30. 10.1084/jem.190.3.323 10430621 PMC2195591

[39] Ehrenstein MR Rada C Jones AM Milstein C Neuberger MS. Switch junction sequences in PMS2-deficient mice reveal a microhomology-mediated mechanism of Ig class switch recombination. Proc Natl Acad Sci U S A (2001) 98:14553–8. 10.1073/pnas.241525998 11717399 PMC64720

[40] Schrader CE Vardo J Stavnezer J. Role for mismatch repair proteins Msh2, Mlh1, and Pms2 in immunoglobulin class switching shown by sequence analysis of recombination junctions. J Exp Med (2002) 195:367–73. 10.1084/jem.20011877 11828012 PMC2193596

[41] Martomo SA Yang WW Gearhart PJ. A role for Msh6 but not Msh3 in somatic hypermutation and class switch recombination. J Exp Med (2004) 200:61–8. 10.1084/jem.20040691 15238605 PMC2213309

[42] Shen HM Tanaka A Bozek G Nicolae D Storb U. Somatic hypermutation and class switch recombination in Msh6(-/-)Ung(-/-) double-knockout mice. J Immunol (2006) 177:5386–92. 10.4049/jimmunol.177.8.5386 17015724

[43] Schanz S Castor D Fischer F Jiricny J. Interference of mismatch and base excision repair during the processing of adjacent U/G mispairs may play a key role in somatic hypermutation. Proc Natl Acad Sci U S A (2009) 106:5593–8. 10.1073/pnas.0901726106 19307563 PMC2659717

[44] Chahwan R Edelmann W Scharff MD Roa S. Mismatch-mediated error prone repair at the immunoglobulin genes. Biomed Pharmacother (2011) 65:529–36. 10.1016/j.biopha.2011.09.001 22100214 PMC3235044

[45] Arkwright PD Gennery AR. Ten warning signs of primary immunodeficiency: a new paradigm is needed for the 21st century. Ann N Y Acad Sci (2011) 1238:7–14. 10.1111/j.1749-6632.2011.06206.x 22129048

[46] Farmand S Baumann U Von Bernuth H Borte M Foerster-Waldl E Franke K [Interdisciplinary AWMF guideline for the diagnostics of primary immunodeficiency]. Klin Padiatr (2011) 223:378–85. 10.1055/s-0031-1287835 22052638

[47] Karaca NE Durandy A Gulez N Aksu G Kutukculer N. Study of patients with hyper-IgM type IV phenotype who recovered spontaneously during late childhood and review of the literature. Eur J Pediatr (2011) 170:1039–47. 10.1007/s00431-011-1400-2 21274562

[48] Murray JE Van Der Burg M IJspeert H Carroll P Wu Q Ochi T Mutations in the NHEJ component XRCC4 cause primordial dwarfism. Am J Hum Genet (2015) 96:412–24. 10.1016/j.ajhg.2015.01.013 25728776 PMC4375537

[49] Picard C Al-Herz W Bousfiha A Casanova JL Chatila T Conley ME Primary immunodeficiency diseases: an update on the classification from the international union of immunological societies expert committee for primary immunodeficiency 2015. J Clin Immunol (2015) 35(8):696–726. 10.1007/s10875-015-0201-1 26482257 PMC4659841

[50] Plaschke J Linnebacher M Kloor M Gebert J Cremer FW Tinschert S Compound heterozygosity for two MSH6 mutations in a patient with early onset of HNPCC-associated cancers, but without hematological malignancy and brain tumor. Eur J Hum Genet (2006) 14:561–6. 10.1038/sj.ejhg.5201568 16418736

[51] Rahner N Hoefler G Hogenauer C Lackner C Steinke V Sengteller M Compound heterozygosity for two MSH6 mutations in a patient with early onset colorectal cancer, vitiligo and systemic lupus erythematosus. Am J Med Genet A (2008) 146A:1314–9. 10.1002/ajmg.a.32210 18409202

[52] Sperotto F Cuffaro G Brachi S Seguso M Zulian F. Prevalence of antinuclear antibodies in schoolchildren during puberty and possible relationship with musculoskeletal pain: a longitudinal study. J Rheumatol (2014) 41:1405–8. 10.3899/jrheum.130948 24737914

[53] Durandy A. Activation-induced cytidine deaminase: a dual role in class-switch recombination and somatic hypermutation. Eur J Immunol (2003) 33:2069–73. 10.1002/eji.200324133 12884279

[54] Povysil G Tzika A Vogt J Haunschmid V Messiaen L Zschocke J panelcn.MOPS: copy-number detection in targeted NGS panel data for clinical diagnostics. Hum Mutat (2017) 38:889–97. 10.1002/humu.23237 28449315 PMC5518446

[55] Etzler J Peyrl A Zatkova A Schildhaus HU Ficek A Merkelbach-Bruse S RNA-based mutation analysis identifies an unusual MSH6 splicing defect and circumvents PMS2 pseudogene interference. Hum Mutat (2008) 29:299–305. 10.1002/humu.20657 18030674

[56] Wernstedt A Valtorta E Armelao F Togni R Girlando S Baudis M Improved multiplex ligation-dependent probe amplification analysis identifies a deleterious PMS2 allele generated by recombination with crossover between PMS2 and PMS2CL. Genes Chromosomes Cancer (2012) 51:819–31. 10.1002/gcc.21966 22585707 PMC3398144

[57] den Dunnen JT Antonarakis SE. Mutation nomenclature extensions and suggestions to describe complex mutations: a discussion. Hum Mutat (2000) 15:7–12. 10.1002/(SICI)1098-1004(200001)15:1<7::AID-HUMU4>3.0.CO;2-N 10612815

[58] Monteiro J Hingorani R Choi IH Silver J Pergolizzi R Gregersen PK. Oligoclonality in the human CD8+ T cell repertoire in normal subjects and monozygotic twins: implications for studies of infectious and autoimmune diseases. Mol Med (1995) 1:614–24. 8529128 PMC2229970

[59] van Dongen JJ Langerak AW Bruggemann M Evans PA Hummel M Lavender FL Design and standardization of PCR primers and protocols for detection of clonal immunoglobulin and T-cell receptor gene recombinations in suspect lymphoproliferations: report of the BIOMED-2 concerted action BMH4-CT98-3936. Leukemia (2003) 17:2257–317. 10.1038/sj.leu.2403202 14671650

[60] Tiller T Meffre E Yurasov S Tsuiji M Nussenzweig MC Wardemann H. Efficient generation of monoclonal antibodies from single human B cells by single cell RT-PCR and expression vector cloning. J Immunol Methods (2008) 329:112–24. 10.1016/j.jim.2007.09.017 17996249 PMC2243222

[61] Berkowska MA Schickel JN Grosserichter-Wagener C De Ridder D Ng YS Van Dongen JJ Circulating human CD27-IgA+ memory B cells recognize bacteria with polyreactive Igs. J Immunol (2015) 195:1417–26. 10.4049/jimmunol.1402708 26150533 PMC4595932

[62] IJspeert H Van Schouwenburg PA Van Zessen D Pico-Knijnenburg I Stubbs AP Van Der Burg M. Antigen receptor galaxy: a user-friendly, web-based tool for analysis and visualization of T and B cell receptor repertoire data. J Immunol (2017) 198:4156–65. 10.4049/jimmunol.1601921 28416602 PMC5421304

[63] Alamyar E Duroux P Lefranc MP Giudicelli V IMGT((R)) tools for the nucleotide analysis of immunoglobulin (IG) and T cell receptor (TR) V-(D)-J repertoires, polymorphisms, and IG mutations: IMGT/V-QUEST and IMGT/HighV-QUEST for NGS. Methods Mol Biol (2012) 882:569–604. 10.1007/978-1-61779-842-9_32 22665256

[64] Gupta NT Vander Heiden JA Uduman M Gadala-Maria D Yaari G Kleinstein SH. Change-O: a toolkit for analyzing large-scale B cell immunoglobulin repertoire sequencing data. Bioinformatics (2015) 31:3356–8. 10.1093/bioinformatics/btv359 26069265 PMC4793929

