# Supplementary material for: No Overt Clinical Immunodeficiency Despite Immune Biological Abnormalities in Patients With Constitutional Mismatch Repair Deficiency
Source: Front Immunol. 2018 Jul 2;9:1506. doi: 10.3389/fimmu.2018.01506 (PMC6036136; doi:10.3389/fimmu.2018.01506)
Supplement: Supplementary file 1 [file Data_Sheet_1.docx]

***Supplementary Material***

**No overt clinical immunodeficiency despite immune biological abnormalities in patients with constitutional mismatch repair deficiency (CMMRD)**

*Tesch VK^1^, IJspeert H^2^, Raicht A^1^, Rueda D^3^, Dominguez-Pinilla N^4^, Allende L.M.^5^, Colas C^6^, Rosenbaum T^7^, Ilencikova D^8^, Baris HN^10^, Nathrath M^11^, Suerink M^12^, Januszkiewicz-Lewandowska D^13^, Ragab I^14^, Azizi AA^15^, Wenzel SS ^16^, Zschocke J ^16^, Schwinger W^1^, Kloor M^17^, Blattmann C^18^, Brugieres L^19^, vanderBurg M^2^, Wimmer K^16^*, and Seidel MG^1, 20^*.*

**, these authors contributed equally to the study.*

*^1-20^See main document for affiliations and correspondence.*

*Supplementary Data: None*

*Supplementary Figures: 1*

*Supplementary Tables: 2*

1. ***Supplementary Figures and Tables***
   1. ***Supplementary Figures***

**Supplementary Figure 1.**

**
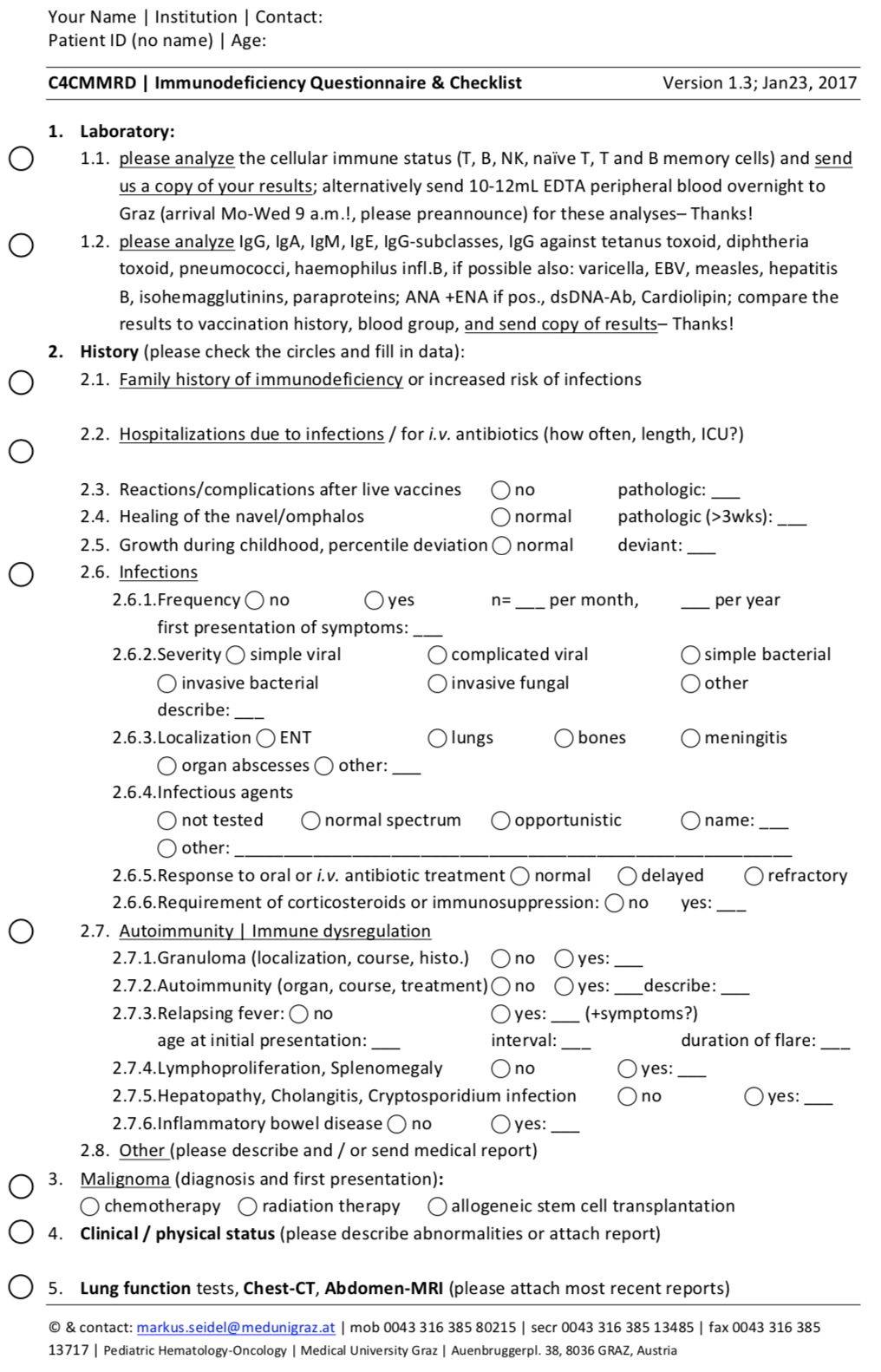
**

- 1. ***Supplementary Tables***
     1. ***Supplementary Table 1. Quantitative analysis of specific antibodies of 9 patients with CMMRD.***

| Parameter (units)  *(reference range after vaccination or infection*)^#^ | P1 | P2 | | P3 | | P5 | P6 | P7 | P11 | P13 | P14 | P16 |
| --- | --- | --- | --- | --- | --- | --- | --- | --- | --- | --- | --- | --- |
| anti-Diphtheria-toxin-Ab (IU/mL)  *(>0.1)* | neg. | 0.22 | | | n.d. | n.d. | n.d. | 0.16 | n.d. | n.d. | 0.05 | 0.05 |
| anti-Tetanustoxin-Ab (IU/mL)  *(>0.1)* | neg. | 0.29 | n.d. | | | n.d. | n.d. | 0.19 | n.d. | n.d. | 0.03 | 3.15 |
| VZV IgG (mU/mL)  *(> 60)* | neg. | 132 | n.d. | | | pos. | n.d. | neg. | n.d. | n.d. | pos. | n.d. |
| EBV (VCA) IgG (U/ml)  *(> 9)* | n.d. | neg. | pos. | | | n.d. | n.d. | neg. | n.d. | neg. | pos. | n.d. |
| CMV IgG (U/mL)  *(>15)* | pos. | n.d. | pos. | | | neg. | n.d. | n.d. | n.d. | n.d. | pos. | n.d. |
| HBS-Ab (IU/L)  *(>100)* | neg. | 150 | n.d. | | | neg. | 146.58 | neg. | n.d. | >100 | n.d.* | n.d. |
| Morbilli IgG (U/mL)  *(>0.15)* | neg. | 169 | n.d. | | | neg. | n.d. | n.d. | n.d. | n.d. | n.d. | n.d. |
| Rubeola IgG-Ab titer  *(>10)* | 18 | n.d. | n.d. | | | n.d. | n.d. | n.d. | n.d. | n.d. | n.d. | n.d. |
| HiB IgG (mg/L)  *(≥0.15)* | neg. | 4.2 | n.d. | | | n.d. | n.d. | <0.11 | <0.11 | n.d. | 1.93 | 2.31 |
| Pneumococci IgG (mg/L)  *(>15-270)* | pos. | 14.6 | n.d. | | | n.d. | n.d. | 5 | n.d. | n.d. | 3.8 | 103 |
| Anti-nuclear Antibodies (ANA)  *(negative when ≤1:80)* | n.d. | neg. | n.d. | | | n.d. | pos. | neg. | pos. | neg. | n.d. | n.d. |

** P14 has chronic Hepatitis C and is anti-HCV IgG positive (not shown), furthermore suffered from parvovirus B19-related erythroblastophthisis after chemotherapy;*

*#, although requested, vaccination and infection histories were unavailable from most patients; isoagglutinin analysis was only performed in two patients (normal, not shown).*

- - 1. ***Supplementary Table 2. Number of unique IGHG and IGHA rearrangements.***

| **CMMRD patients** | **age** | **number of IGHG rearrangements** | **number of IGHA rearrangements** |
| --- | --- | --- | --- |
| P11 | 7 | 263 | 305 |
| P16 | 8 | 239 | 255 |
| P5 | 10 | 121 | 132 |
| P14 | 7 | 178 | 139 |
| P13 | 10 | 198 | 85 |
| **Controls** | **age** | **number of IGHG rearrangements** | **number of IGHA rearrangements** |
| NWK56 | 6 | 101 | 200 |
| NWK64 | 7 |  | 69 |
| NWK61 | 8 |  | 55 |
| NWK66 | 9 | 47 | 76 |
| NWK57 | 14 | 197 | 177 |
| NWK42 | 15 | 130 | 106 |
| NWK5 | 15 |  | 103 |
| NWK53 | 18 | 423 | 405 |
| NWK43 | 20 | 224 | 221 |
| NWK303 | 22 | 545 | 812 |
